# An integrative metabolomics and transcriptomics study to identify metabolic alterations in aged skin of humans *in vivo*

**DOI:** 10.1186/s12864-017-3547-3

**Published:** 2017-02-15

**Authors:** Andreas Kuehne, Janosch Hildebrand, Joern Soehle, Horst Wenck, Lara Terstegen, Stefan Gallinat, Anja Knott, Marc Winnefeld, Nicola Zamboni

**Affiliations:** 1 Institute of Molecular Systems Biology, ETH Zurich, Auguste-Piccard-Hof 1, 8093 Zürich, Switzerland; 2 PhD Program Systems Biology, Life Science Zurich Graduate School, Zurich, Switzerland; 3 Beiersdorf AG, R&D, Skin Research Center, Unnastrasse 48, Hamburg, 20253 Germany; 4 Coburg University of Applied Sciences and Arts, Friedrich-Streib-Straße 2, Coburg, 96450 Germany

**Keywords:** Skin, Aging, Metabolism, Metabolomics, Transcriptomics, Systems biology

## Abstract

**Background:**

Aging human skin undergoes significant morphological and functional changes such as wrinkle formation, reduced wound healing capacity, and altered epidermal barrier function. Besides known age-related alterations like DNA-methylation changes, metabolic adaptations have been recently linked to impaired skin function in elder humans. Understanding of these metabolic adaptations in aged skin is of special interest to devise topical treatments that potentially reverse or alleviate age-dependent skin deterioration and the occurrence of skin disorders.

**Results:**

We investigated the global metabolic adaptions in human skin during aging with a combined transcriptomic and metabolomic approach applied to epidermal tissue samples of young and old human volunteers. Our analysis confirmed known age-dependent metabolic alterations, e.g. reduction of coenzyme Q10 levels, and also revealed novel age effects that are seemingly important for skin maintenance. Integration of donor-matched transcriptome and metabolome data highlighted transcriptionally-driven alterations of metabolism during aging such as altered activity in upper glycolysis and glycerolipid biosynthesis or decreased protein and polyamine biosynthesis. Together, we identified several age-dependent metabolic alterations that might affect cellular signaling, epidermal barrier function, and skin structure and morphology.

**Conclusions:**

Our study provides a global resource on the metabolic adaptations and its transcriptional regulation during aging of human skin. Thus, it represents a first step towards an understanding of the impact of metabolism on impaired skin function in aged humans and therefore will potentially lead to improved treatments of age related skin disorders.

**Electronic supplementary material:**

The online version of this article (doi:10.1186/s12864-017-3547-3) contains supplementary material, which is available to authorized users.

## Background

Tissue aging is caused by intrinsic and extrinsic factors that induce complex molecular changes and, in turn, a deterioration of cellular structures and function. These changes are major causes of age-related diseases like cancer or cardiovascular disorders [1, 2]. The main molecular adaptations occurring during aging are loss of genomic stability due to reduced DNA repair capacities [3], loss of proliferative potential caused by increased senescence [1, 4], and age-related alterations in the DNA-methylation patterns that affect cellular plasticity [5, 6]. Metabolic adaptations are also considered to play a major role in aging [7–10]. For instance, the metabolic function of mitochondria is progressively impaired during aging in different tissues [8, 11]. This can result in increased generation of reactive oxygen species that foster genomic instability [8, 12]. Moreover, several studies reported that caloric restrictions and diet adaptations, such as supplementation of food with branched chain amino acids [13, 14], can significantly increase lifespan [15]. This suggests that metabolic activity as well as nutrient sensing pathways are highly relevant for cellular aging processes (reviewed in [10]). Accordingly, interference with the insulin/IGF1 and the mammalian target of rapamycin (mTOR) pathways increased lifespan in different model organisms [7, 16–18].

While the underlying molecular mechanisms that cause cellular aging and influence lifespan of model organisms are well described, the mechanistic details of age-related alterations in human tissues *in vivo* are barely explored. This is due to the low availability of healthy human tissue samples from internal organs of donors of different age [19]. Skin is an exception because it’s simply accessible and thus constitutes a good model to study aging in humans [20]. Skin aging is caused by both intrinsic factors including age-dependent changes in hormonal levels and extrinsic factors, such as smoking and UV exposure. Both intrinsic and extrinsic factors induce significant morphological changes such as wrinkles, reduced elasticity, increased pigmentation and thinning of the epidermis [2, 20–24]. Moreover, metabolic studies suggested that aged epidermal keratinocytes shift their energy generation from aerobic respiration in mitochondria to anaerobic glycolysis. This was attributed to a reduction of coenzyme Q10 levels in the respiratory chain [25–27]. Notably, metabolites such as coenzyme Q10 or vitamins are widely used in anti-aging treatment in skin care products [25, 28–31]. These examples highlight the relevance of metabolic changes in human skin aging, both as drivers of functional deterioration as well as a target for anti-aging treatments.

Besides the reduction in respiratory chain activity, however, very little is known about metabolic alterations in aged skin. Due to the fact that metabolism is crucial to support further skin functions, e.g. the epidermal water loss barrier or epidermal differentiation, we analyzed the global metabolic adaptations occurring in human epidermal skin during aging. We applied an integrative metabolomics and transcriptomics approach on healthy epidermal tissue from young and old human donors. The analysis revealed age-dependent metabolic adaptations of metabolites already reported to be involved in skin aging and metabolites with potential impact on skin function, such as osmolytes. Moreover, the integration of transcriptome and metabolome data revealed a transcriptionally regulated reduction in protein as well as polyamine biosynthesis and adaptation in upper glycolysis and glycerolipid biosynthesis in aged skin.

## Results

### Differences in the epidermal skin metabolome of young and old human volunteers

**Fig. 1:**
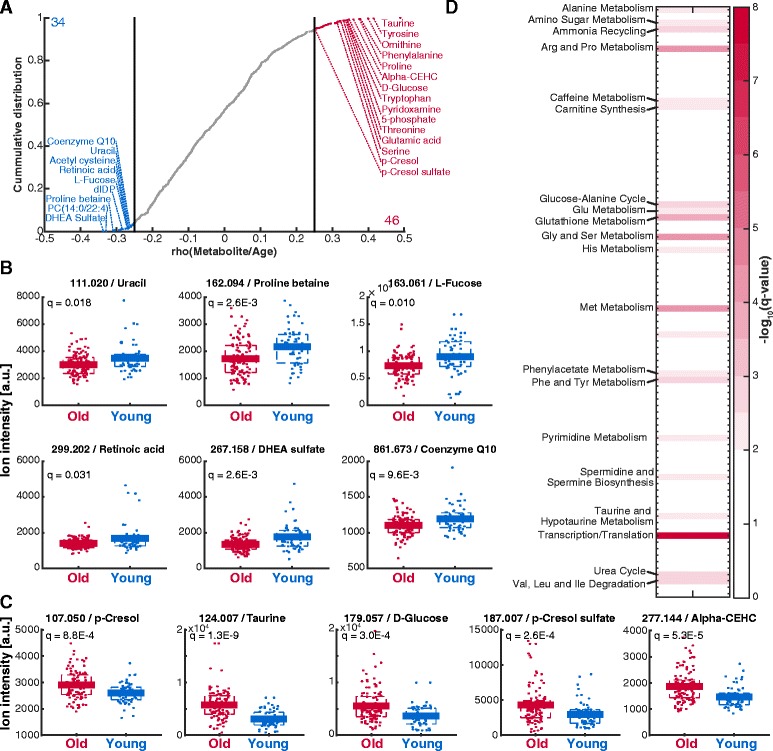
Metabolome differences between young and old human skin. **a** Correlation analysis of metabolites and donor age. Correlations with |rho| > 0.25 and q < 0.01 are considered significant. **b-c** Example of metabolites that either decrease (in **b**) or increase (in **c**) significantly during aging (q < 0.05; differential analysis old vs young). **d** Metabolic pathway enrichment analysis on significantly changing ions obtained selecting metabolites with (|log_2_(fold-change)| > 0.1 and q < 0.05) and the pathways as defined by the Human Metabolome DB (HMDB). Only enriched pathways are listed

To chart metabolic adaptations in human skin during aging *in vivo*, we performed non-targeted metabolomics analysis of epidermal skin tissue samples obtained from the inner side of the forearm of 28 young (20 to 25 years) and 54 old (55 to 66 years) female human donors. Polar metabolite extracts were analyzed by flow injection time-of-flight mass spectrometry as described before (Additional file 1, Additional file 2) [32]. In total we detected 4585 ions of which 829 could be putatively assigned to 2530 metabolites listed in the Human Metabolome Database v3.0 (HMDB) [33] on the basis of accurate mass, isotopologue abundance, and cross-correlation [32]. To account for differences in the amount of epidermal tissue, we normalized the intensities using quantile normalization [34]. To find age related differences in metabolism, we performed two different analyses: On the one hand we correlated metabolite intensities with donor age (Fig. 1a) and on the other hand we performed a univariate analysis to compare metabolite levels in skin of young and old donors (Additional file 3 A). In both analyses, less than 10% of the metabolites indicated significant age-dependent alterations. In the correlation analysis, 34 metabolites negatively correlated and 46 positively correlated with age (Fig. 1a). Comparably, the univariate analysis indicated that the levels of 10 metabolites decreased in old compared to young donors while 46 metabolites increased (Additional file 3 A).

Next, we focused on metabolites with potential relevance for skin function that decreased with advancing age. Consistent with previous studies, coenzyme Q10 levels were lower in epidermis of elderly donors (Fig. 1b). This reduction in coenzyme Q10 is thought to play a major role in impaired mitochondrial function during aging [26, 27]. Moreover, we found metabolites with age-dependent level reduction that are known to feedback to important cellular signaling processes. For instance, retinoic acid was found lower in aging skin and is involved in the regulation of keratinocyte proliferation and differentiation during epidermal homeostasis (Fig. 1b) [35]. Additionally, we found an age-dependent decrease of the hormone dehydroepiandrosterone (DHEA) sulfate (Fig. 1b). It is known that the blood levels of DHEA and its conjugate DHEA sulfate decrease with age [36]. Our study suggests that this age-dependent reduction of the systemic DHEA availability translates to the *in vivo* concentration in human epidermis. Furthermore, we observed an age-dependent change in the concentration of organic osmolytes, which convey protection against environmental stresses, for instance ultraviolet radiation, in human skin [37–39]. We measured a reduction of the organic osmolyte proline betaine and increased levels of taurine, which are involved osmoprotection of human skin cells (Fig. 1b) [39–42]. With an average 1.8-fold increase, taurine was the largest change in the metabolome (Additional file 3 A). Besides taurine, other metabolites with potential relevance for skin function were found to be increased such as for example the aging biomarker candidates cresol and cresol sulfate [43] and the vitamin E metabolite α-CEHC (Fig. 1c). Vitamin E metabolites carry important anti-oxidative functions in skin and protect against oxidative damage caused by UV irradiation [44]. Besides these alterations glucose levels were also increased in aged skin (Fig. 1c). Previous studies showed that glucose uptake is elevated *in vitro* in cultured keratinocytes from old compared to those from young donors [25]. It is thought that the major part of the additionally taken up glucose is converted to lactate potentially to compensate energy deficits due to defects in mitochondrial respiration. Thus, the increased glucose levels in aged skin might indicate that the increased glucose uptake is also relevant in human epidermis *in vivo*.

**Fig. 2:**
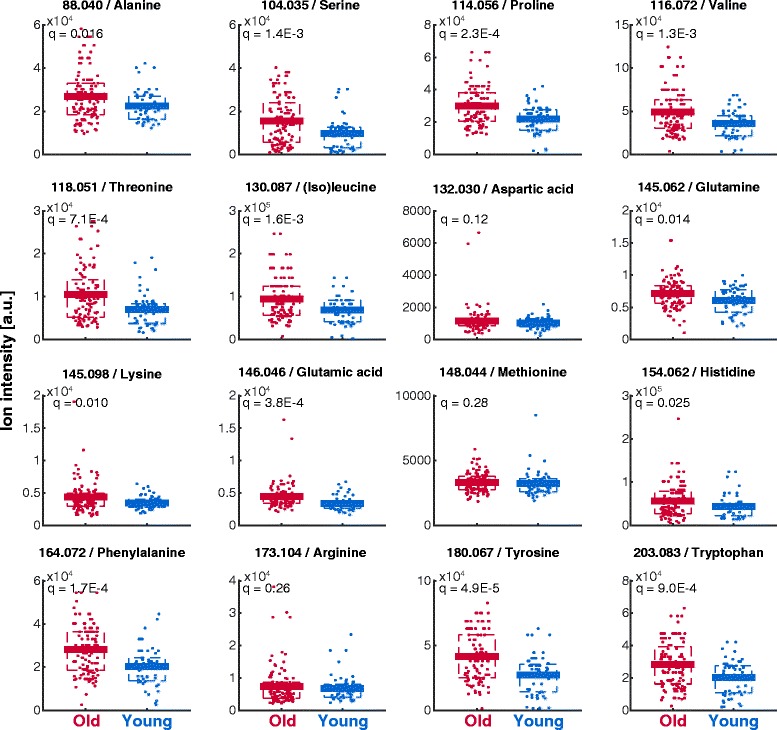
Differences in amino acid metabolite levels comparing young and old skin. The number in the title reports the measured m/z. The q-values are FDR-corrected p-values obtained from unpaired, heteroscedastic t-test

To elucidate if aging induced milder but accumulated metabolic adaptations in specific metabolic pathways, we performed a pathway enrichment analysis on the basis of significantly changing metabolite ions (Additional file 3 B). We found an enrichment for amino sugar metabolism, ammonia recycling, glutathione metabolism, mitochondrial electron transport chain, urea cycle and different amino acid metabolism pathways including arginine and proline metabolism, glycine and serine metabolism, methionine metabolism and transcription/translation (Fig. 1d). In agreement, the metabolite levels of most amino acids increased with age (Figs. 1a and 2). The general accumulation of amino acids might be the mere consequence of decreased protein biosynthesis associated to the reduced proliferation in aged skin. Alternatively, amino acids are natural moisturizing factors and their increase might reflect an adaptive response to prevent skin dryness in the epidermis of elder humans [45].

### Age-dependent adaptations of gene expression in epidermal skin

**Fig. 3:**
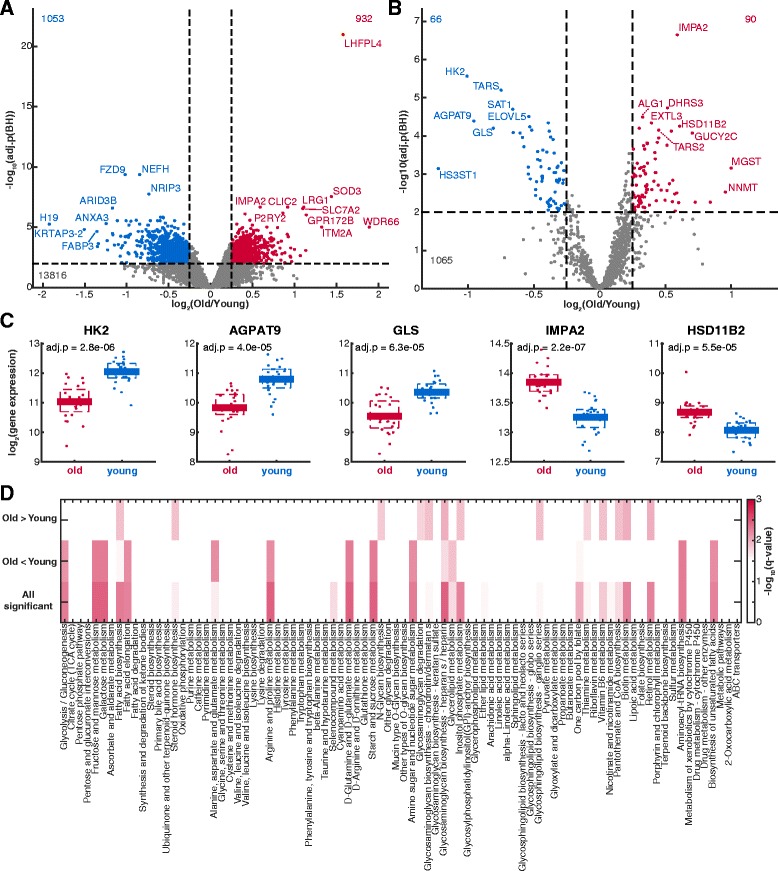
Transcriptional differences between young and old human skin. **a-b** Significant changes in gene expression of either all (**a**) or only metabolic enzyme coding genes (**b**). **c** Examples of metabolic enzymes with significantly changing gene expression. Adjusted p-values (adj.p) in **a-c**) are false discovery corrected *p*-values using the from a differential analysis. **d** Pathway enrichment analysis of significantly changing transcripts. Q-values are FDR-corrected p-values of a hypergeometric test

We performed a complementary transcriptome analysis using Agilent Whole Human Genome Oligo Microarrays 8x60K V2 on epidermal tissue samples from 24 young and 24 old donors, of which 23 donors of each group were also included in the metabolomics analysis (Additional file 1). In total, 1053 transcripts indicated significant decreased and 932 transcripts significant increased levels (Fig. 3a). We compared the identified age-dependent genes of our study with genes predicted to be involved in aging in humans in multiple tissues [46] and with genes classified to show age-dependent changes in gene expression in skin [19, 47]. The identified genes with age-dependent expression of our study had no significant overlap with any of the age-dependent gene groups from the other studies (p > 0.05, hypergeometric-test, Additional file 4). However, also a comparison amongst the reported genes with age-dependent expression of the different studies demonstrated no significant overlap (Additional file 4). This indicated that age-dependent gene expression strongly depends on the originating tissue. Furthermore, age-dependent gene expression in skin likely depends on the anatomic location of the sample and on the composition of the skin tissue, e.g. epidermis, dermis, or subcutaneous tissue [48].

To investigate the potential role of altered expression of genes encoding for metabolic enzymes in mediating metabolic adaptations during aging, we focused on the 1140 transcripts that could be mapped to genes involved in human metabolism according to the KEGG database (Fig. 3b) [49]. Notably, similar to the metabolic alterations, the transcript level changes of metabolic enzymes are only mild and with one exception do not exceed a two-fold increase or decrease in expression (Fig. 3b). In total, 66 metabolic enzyme encoding genes demonstrated a reduced expression and 90 an elevated expression (Benjamini-Hochberg adjusted *p* < 0.01, |log_2_(fold change)| > 0.25, Fig. 3b). Among the genes with the strongest reduction in expression we found hexokinase 2 (HK2) and glutaminase (GLS), which were both reported to be essential for energy generation to support proliferation in different cancer types [50, 51]. Therefore, the age-dependent decrease of those enzymes could be related to reduced proliferation of epidermal cells during aging [52]. In addition, glutaminase converts glutamine into glutamate, which is involved in the homeostasis of the epidermal barrier [53] (Fig. 3c). Moreover, we identified enzymes with larger changes in expression that are involved in keratinocyte differentiation. For example, the glycerol-3-phosphate acyltransferase 3 (AGPAT9) showed almost 50% reduction in gene expression during aging (Fig. 3c). This enzyme is involved in the synthesis of glycerolipids that are essential for the formation of the epidermal barrier [54, 55]. If this age-dependent decrease of the enzyme is functional and results in reduced glycerolipid biosynthesis, it might be involved in impaired epidermal barrier formation in the *stratum corneum* of aged skin [28]. The expression of inositol-1(or 4)-monophosphatase 2 (IMPA2) that is involved in inositol phosphate metabolism and hydroxysteroid (11-beta) dehydrogenase 2 (HSD11B2), which is involved in cortisol homeostasis, were elevated in the skin of old donors (Fig. 3c). Previous studies showed that both metabolic systems adapt during differentiation and were involved in regulation of epidermal homeostasis [56–59].

To elucidate the metabolic pathways that are effected by accumulated adaptations in gene expression, we performed a pathway enrichment analysis on transcriptome data using the KEGG pathway definition [49]. Several metabolic pathways involved in keratinocyte differentiation showed a significant enrichment (Fig. 3d), for instance inositol phosphate metabolism with generally elevated gene expression (Additional file 5 B) and retinol metabolism with a mix of increased and decreased gene expression (Additional file 5 E). We additionally identified increased expression of enzymes in different pathways including glycosaminoglycan biosynthesis, steroid hormone biosynthesis or pantothenate and CoA metabolism (Fig. 3d). In contrast, pathways involved in central carbon metabolism, in amino acid metabolism (e.g. arginine and proline metabolism), tRNA biosynthesis or amino- and nucleotidesugar metabolism were enriched for enzymes with decreasing gene expression during aging (Fig. 3d, Additional file 5 ACD). In summary, we identified age-dependent changes in gene expression in different metabolic pathways that have been associated with epidermal homeostasis and therefore might be important to sustain epidermal function.

### Integrated analysis of transcriptome and metabolome data

Since the age-dependent adaptations of metabolite and transcript levels are only mild, we set out to identify metabolic enzymes that featured an age-dependent and functional change in activity driven by altered gene expression. We hypothesized that functional changes in expression of enzyme encoding genes should induce alterations of the levels of proximal metabolites. We applied a previously developed locality scoring approach [60] on the matched transcriptome and metabolome data of 23 young and 23 old donors (Additional file 1). The algorithm assumes that a functional change in enzyme levels should induce (anti)correlating adaptations in the substrates and products of the catalyzed reaction. It scores each enzyme by a weighted sum of the correlation of the enzyme’s gene expression and the intensities of surrounding metabolites.

We found 61 enzymes with significant locality scores suggesting that altered gene expression had a functional impact on metabolic activity (Additional file 6). To infer which of these functional hits mediate age-dependent metabolic alterations, we focused on the 21 predicted enzymes with age-dependent gene expression (Table 1). Amongst the top hits were the aldehyde dehydrogenase 4 family, member A1 (ALDH4A1, Additional file 7) and the branched chain keto acid dehydrogenase (BCKDHA, Additional file 8). Moreover, interleukin 4 induced 1 (IL4I1), which is a lysosomal amino-acid oxidase that decreased in aged skin, had a significant locality score (Additional file 9). The lower expression of this amino acid oxidase potentially explains at least partially the increased levels of amino acids, like phenylalanine or tyrosine, and the reduced levels of their oxidation products, like 2-hydroxypheylacetate or homogentisate, in aged epidermis (Additional file 9).

**Table 1:** Results of locality analysis of genes with changing transcript levels over time

Gene Symbol	Gene Name	Locality		Correlation Gene/Age		Diff. Analysis Old/young	
		Score	p	r	p	log2 (FC)	adj.p
ALDH4A1	aldehyde dehydrogenase 4 family, member A1	0.25	1E-04	−0.33	0.026	−0.26	0.006
ODC1	ornithine decarboxylase 1	0.26	2E-04	−0.26	0.076	−0.30	0.085
GALNT6	polypeptide N-acetylgalactosaminyltransferase 6	0.22	4E-04	0.38	0.01	0.30	0.112
TARS2	threonyl-tRNA synthetase 2, mitochondrial (putative)	0.31	7E-04	0.55	7E-05	0.45	7E-05
BCKDHA	branched chain keto acid dehydrogenase E1, alpha polypeptide	0.21	0.002	0.34	0.022	0.18	0.026
ALDOA	aldolase A, fructose-bisphosphate	0.24	0.003	0.57	3E-05	0.24	0.002
TARS	threonyl-tRNA synthetase	0.29	0.003	−0.54	1E-04	−0.75	6E-06
YARS2	tyrosyl-tRNA synthetase 2, mitochondrial	0.28	0.006	−0.39	0.007	−0.27	0.011
CYP51A1	cytochrome P450, family 51, subfamily A, polypeptide 1	0.19	0.008	−0.40	0.005	−0.20	0.012
ALAD	aminolevulinate dehydratase	0.24	0.008	0.19	0.204	0.22	0.061
IL4I1	interleukin 4 induced 1	0.21	0.014	−0.16	0.297	−0.58	0.052
FBP1	fructose-1,6-bisphosphatase 1	0.27	0.016	0.34	0.022	0.22	0.094
GATM	glycine amidinotransferase (L-arginine:glycine amidinotransferase)	0.26	0.017	0.37	0.012	0.29	0.019
YARS	tyrosyl-tRNA synthetase	0.27	0.02	−0.48	8E-04	−0.54	6E-04
GART	phosphoribosylglycinamide formyltransferase, phosphoribosylglycinamide synthetase, phosphoribosylaminoimidazole synthetase	0.28	0.021	−0.35	0.019	−0.39	0.006
PRDX6	peroxiredoxin 6	0.24	0.021	0.36	0.015	0.14	0.056
AGA	aspartylglucosaminidase	0.18	0.032	0.25	0.101	0.15	0.077
TST	thiosulfate sulfurtransferase (rhodanese)	0.24	0.034	0.31	0.039	0.31	0.041
ACP5	acid phosphatase 5, tartrate resistant	0.26	0.038	0.31	0.036	0.36	0.007
IARS	isoleucyl-tRNA synthetase	0.28	0.039	−0.40	0.006	−0.44	0.002
NDUFV2	NADH dehydrogenase (ubiquinone) flavoprotein 2, 24kDa	0.22	0.041	0.26	0.077	0.13	0.062

All genes with *p* < 0.05 for the locality analysis and *p* < 0.1 for the gene-age correlation or the differential analysis are listed. The full results are summarized in Additional file 6

**Fig. 4:**
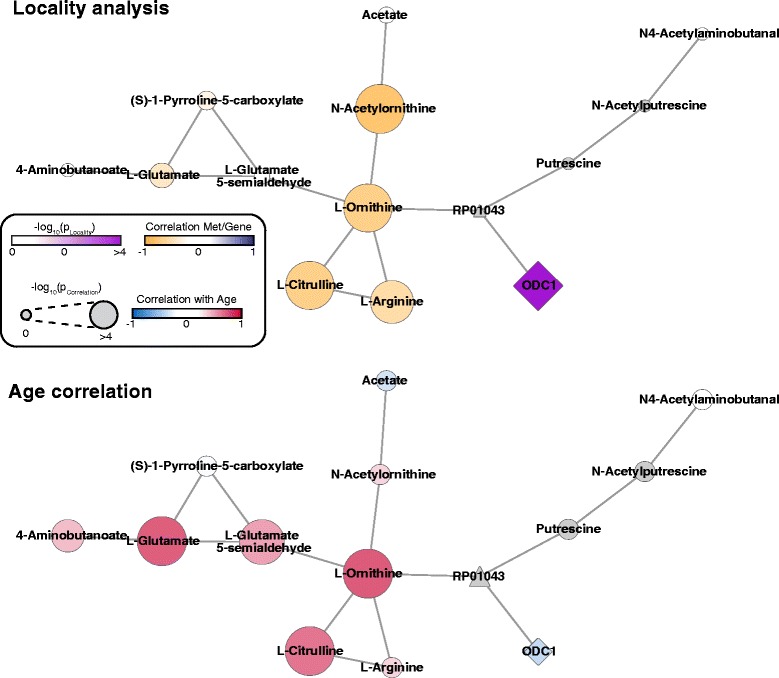
Change of tRNA synthetases and amino acid levels during aging. Q-values are FDR-corrected p-values of a t-test comparing metabolite intensities of old and young skin samples. Stars (*) mark genes with significant changes in gene expression comparing skin from old and young donors (|log_2_(old/young)| > 0.25, adj.*p* < 0.01)

Next we concentrated on three cases that might be relevant for age-dependent skin defects: i) amino acid tRNA synthetases, ii) polyamine biosynthesis, and iii) switch between glycolysis and glycerolipid metabolism. Several amino acid tRNA synthetases indicated a significant locality score and age-dependent gene expression (Table 1). Both the increase of amino acid metabolite levels and change in tRNA synthetase expression with age emerged already in the individual analysis of metabolome and transcriptome (Fig. 2, Additional file 5 C). We identified five amino acid - tRNA synthetase pairs with significantly decreased gene expression of the tRNA synthetases and elevated levels of the corresponding amino acids in aged skin (Fig. 4). This suggests that protein biosynthesis is - like in other organisms and tissues [61] - reduced in old skin, which could be linked to a lower proliferation in the epidermis [52].

**Fig. 5:**
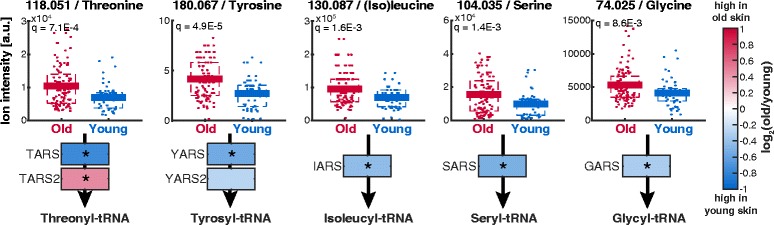
Age-dependent changes in ornithine utilization. *Upper panel*: Locality scores for and metabolite-gene correlation. *Lower panel*: Metabolite-age and gene-age correlation. Node size indicates statistical significance

The ornithine decarboxylase 1 (ODC1) indicated a decreased gene expression in aged skin and was amongst the top hits of the locality scoring (Table 1, Fig. 5). ODC1 catalyzes the conversion of ornithine to putrescine, the committing step in the biosynthesis of polyamines. Polyamines are essential to support cell growth and proliferation in normal and cancerous cells [62]. In skin it has been shown that ODC1 gets activated upon UV exposure [63] and is crucially involved in the development of both squamous and basal cell carcinomas [64–66]. Polyamines were reported to decrease during aging in different organism and supplementation of them to an organism’s diet increased live span [67]. Moreover, in rat skin it was shown that ornithine decarboxylase activity decreases with age [68]. Our study suggests that ornithine decarboxylase activity declines also in epidermal skin tissue of humans during aging. However, whether the probable reduction in polyamine biosynthesis is involved in the reduced proliferation in the epidermis still remains open [52].

**Fig. 6:**
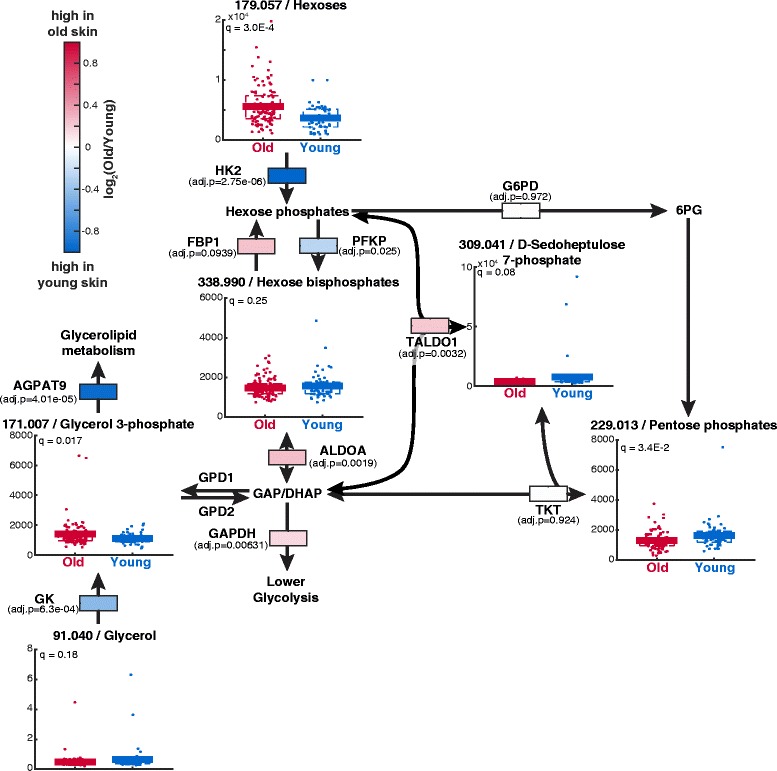
Changes in upper glycolysis, glycerolipid metabolism and pentose phosphate pathway during aging. Q-values are false discovery corrected p-values of a t-test comparing metabolite intensities of old and young skin samples. Adjusted p-values (adj.p) in A-C) are FDR-corrected p-values from a differential analysis

Finally, we elaborate on a last case of age-dependent metabolic adaptions at the interface of upper glycolysis and glycerolipid metabolism. The locality analysis identified the fructose bisphosphatase 1 (FBP1) and aldolase A (ALDOA) as age-dependent enzymes with correlating metabolite changes (Table 1). Moreover, in the transcriptomics analysis AGPAT9, HK2 and glycerol kinase (GK) were amongst the enzymes with the highest magnitude changes (Fig. 3bc). A detailed investigation within the context of the metabolic network indicated that the decreased expression of HK2 might explain the increased glucose (hexose) metabolite pool and the decreased levels of pentose phosphates metabolites including sedoheptulose phosphate and the pentose phosphates in aged skin (Fig. 6). Additionally, the slightly elevated transcript levels of FBP1 as well as ALDOA and the reduced levels of phosphofructokinase (PFKP) could suggest that glycolytic flux is reduced and gluconeogenesis activated (Fig. 6). However, this is contradicted by previous studies reporting that old keratinocytes increase glycolytic flux [25] and also by the slightly increased expression of glyceraldehyde 3-phosphate dehydrogenase (GAPDH, Fig. 6). Another potential explanation could be the link of glycolysis to glycerolipid metabolism. The expression of AGPAT9 and GK, both involved in glycerolipid biosynthesis, was significantly lowered during aging suggesting that glycerolipid biosynthesis is reduced in old skin (Fig. 6). Recent studies suggested that increased glycerolipid biosynthesis during keratinocyte differentiation mediated by elevated AGPAT9 expression is essential for epidermal barrier formation [54, 55]. The reduction in glycerolipid biosynthesis could lead to a reduced necessity of carbons from upper glycolysis in the epidermis of old human donors which would also explain the transcriptional and metabolic adaptations we observed.

## Discussion

Human skin undergoes significant morphological and functional changes during aging, including wrinkle formation, thinning of the epidermis and altered epidermal barrier function [20, 23, 24, 28]. Besides these high-level adaptations, different studies demonstrated that metabolic activity is altered in aged skin as well [25, 26]. In this study we aimed at expanding the knowledge on age-dependent metabolic adaptions in human skin using a combined transcriptomic and metabolomics approach applied on epidermal skin tissue samples of young and old human donors. It should be noted, that we used exclusively skin samples from female volunteers. Although there is no evidence that gender-related genes are affected, we cannot completely rule out this possibility.

Both the metabolomics and transcriptomics analysis revealed that less than 10% of the detectable metabolites and transcripts adapted significantly during aging. Importantly, in comparison to many other studies the magnitudes of the age-dependent metabolite and transcript changes were only minor. From our perspective, this is not surprising, because in contrast to other biological perturbations such as cancerous transformations - that are associated with massive cellular alterations - epidermal cells need to maintain in general the functionality of human skin whether they are young or old. Nevertheless, the minor metabolite as well as expression changes that we have identified in this study could contribute to the morphological and molecular alterations that are associated with skin aging.

**Fig. 7:**
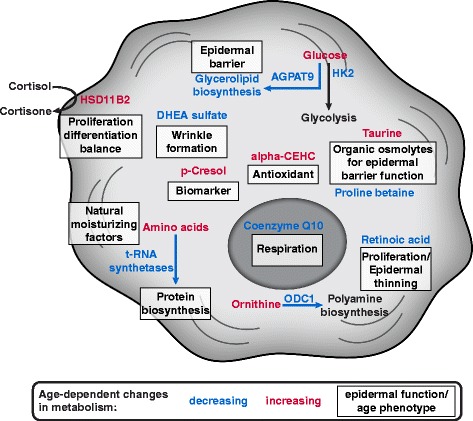
Overview of age-dependent metabolic changes and their potential functional implication in human skin

Due to the mild adaptions, the identification of functionally altered metabolic activity in aged skin interpretation of significant metabolite and transcript changes of small magnitude is especially challenging. Therefore, we employed the previously presented locality scoring approach [60] to identify age-dependent transcriptional alterations of enzymes that functionally effect proximal metabolic activity and thus metabolite levels. This integrated analysis revealed age-dependent, concerted metabolite and transcript changes that are potentially relevant for skin and in particular epidermal function. Additionally, in the individual analysis of the two datasets we identified other adaptations of metabolites and transcripts of high magnitude that are most likely relevant for altered skin function in aged skin. Together, we categorize those alterations into adaptations that potentially effect cellular signaling, epidermal barrier, and skin structure (Fig. 7).

### Feedback of metabolic alterations in aged skin to cellular signaling

The first category of metabolic adaptations includes altered hydrocortisone homeostasis and decrease of retinoic acid metabolite levels during aging. Both are involved in the regulation of proliferation and differentiation in epidermal keratinocytes, which is important for continuous epidermal maintenance (Fig. 7) [35, 57, 58]. Interestingly, recent studies demonstrated that topical treatment with retinoids increased epidermal thickening and also reduced the effects of photoaging [69, 70]. Therefore, the reduction in retinoid levels is potentially involved in the decrease of keratinocyte proliferation and reduction of epidermal thickness during aging [52, 71].

### Age-dependent metabolic adaptations and epidermal barrier function

The second group contains age-dependent metabolic adaptations that might affect epidermal barrier function. These comprise adaptations of the levels of amino acids functioning as natural moisturizing factors [45], of α-CEHC functioning as antioxidant [44, 72] and of proline betaine as well as taurine serving as organic osmolytes [37–39]. Considering epidermal skin function, the latter are of special interest. Skin cells are frequently exposed to environmental stresses, such as UV irradiation or climatic changes, that cause highly varying osmotic pressures [45]. For instance UV radiation induces oxidative stress [73] that causes cell hydration changes and thus hyperosmotic stress [39, 45]. Under these stress conditions, skin cells actively take up organic osmolytes, such as taurine or betaine, to regulate intracellular water levels and counteract cell hydration changes [37–39, 45]. Therefore organic osmolytes are suggested to play a major role in maintaining skin hydration [45]. Additionally, organic osmolytes act as antioxidants that prevent oxidative damage induced by environmental stressors [45]. Furthermore, taurine exhibits anti-apoptotic activity, prevents cell membrane disruption upon UV exposure [45, 74], and stimulates the synthesis of lipids for the epidermal barrier [45, 75]. Interestingly, proline betaine was reduced in aged skin while taurine levels were upregulated. Thus, we hypothesize that the regulation of organic osmolytes appears to be more complex and a balanced interplay of these molecules has to be present to induce positive effects. In aged skin we observed an altered balance comparing proline betaine and taurine levels suggesting that increased taurine levels alone are not sufficient to prevent body dehydration through transepidermal water loss and to protect against environmental stresses like UV irradiation in the thinned aged skin.

Moreover, using our integrative approach we identified age-dependent adaptations at the interface of upper glycolysis and glycerolipid metabolism with potential impact on epidermal barrier function (Fig. 7). On a first sight the metabolite and transcript differences between young and old skin suggest that glycolytic flux is reduced and gluconeogenetic flux increased during aging. However, this contradicts with a previous study in young and old keratinocytes *in vitro,* which reported that old keratinocytes have increased glucose uptake and lactate secretion indicative of increased glycolysis [25]. Since important enzymes for the rerouting of glycolytic flux into glycerolipid metabolism showed a decreased expression during aging, another potential explanation could be that less carbon is needed to fuel glycerolipid metabolism in the epidermis of old donors. This reduction in glycerolipid metabolism could have severe consequence for the lipid layer of the epidermal barrier in the *stratum corneum* and might lead to impaired barrier function in aged skin [54, 76–78].

### Influence of altered metabolism on skin structure in old humans

During aging skin undergoes different structural adaptations including thinning of the epidermis due to reduced proliferation of epidermal keratinocytes [52]. We identified different age-dependent metabolic adaptations that might be involved in this phenotype. For example, in the integrated transcriptome and metabolome analysis, we observed a reduction in tRNA synthetase levels linked to an increase in the levels of their corresponding amino acids (Fig. 7). This adaptation might be due to a reduced protein synthesis in the epidermis of old donors. Multiple studies reported that protein turnover rate, i.e. protein synthesis and protein degradation, is diminished during aging in various organisms and that an artificial reduction of protein synthesis prolongs lifespan (Reviewed in [61]). We argue that protein biosynthesis decreases during aging in human epidermis as well. This might be cause or consequence of a reduction in proliferation of epidermal keratinocytes [52]. Additionally, the integrated analysis revealed a second age-dependent adaptation in polyamine biosynthesis mediated by ODC1 that also could influence proliferation in the epidermis (Fig. 7) [67, 68]. Polyamines are necessary for cell growth and proliferation [62] and therefore we suggest that the reduction in ODC1 transcript levels during aging causes a reduced polyamine biosynthesis which could also cause the reduced proliferation in the aged skin [52]. Besides epidermal thinning, aged skin is also less elastic and forms wrinkles. In the metabolomics analysis we identified decreased DHEA sulfate levels in epidermal samples from old donors, which potentially has an influence on these structural properties of the skin. DHEA sulfate is a human hormone with reported age-dependent reduction of its levels in the blood [36]. Our study suggests that this age-dependent decrease in DHEA availability is translated to the *in vivo* concentration in the epidermis. DHEA and DHEA sulfate regulate collagen synthesis and matrix metalloproteinase (MMP) production in the dermis [79–81], which are both causing mechanical defects in aged skin including wrinkling and loss of elasticity [22, 81, 82]. Though we did measure the DHEA sulfate levels only in the epidermis, we propose that DHEA sulfate levels decrease in the whole skin tissue during aging *in vivo*. Probably, they are mediating alterations in the collagen network in the dermis that account for the changed mechanical properties of aged skin. Indeed, different studies demonstrated that topical treatment with DHEA induced collagen synthesis as well as decreased MMP levels in aged skin and oral treatment improved skin status of old humans [36, 81].

## Conclusion

The integrated metabolome and transcriptome analyses on human epidermal tissue samples provide an overview of the global metabolic adaptations in epidermal skin during aging and their potential impact on skin function. Considering that different metabolites, including coenzyme Q10 [83], α-CEHC [72] or DHEA sulfate [36, 81], are able to reverse age related changes in human skin and are therefore included in anti-aging skin care products, this knowledge will be valuable to improve skin care and treatments of age-related skin disorders like xerosis [84].

## Methods

### Collection of skin tissue samples

Suction blistering is a technic that can be used to separate epidermis from dermis by purely mechanical forces avoiding chemical or thermal damage [85]. Epidermis samples (suction blister roofs) were obtained as described previously from the inner forearms of 28 young (20 to 25 years) and 54 old (aged between 55 and 66 years) healthy female volunteers [86]. Epidermis samples were taken and immediately stored at -80°C. For metabolomics analysis epidermis samples were lysed in 350 μL isopropanol utilizing a Precellys 24 homogenizer (Peqlab). For transcriptomics analysis the epidermis samples were homogenized using a ball mill (MM301, Retsch and TissueLyser Adapter Set, Qiagen).

### Non-targeted metabolomics analysis

For mass spectrometric analysis intracellular samples were analyzed in undiluted or in a 1-10 dilution in ddH_2_O, and extracellular samples were analyzed with a 1-20 dilution in ddH_2_O by flow injection analysis on an Agilent Q-TOF 6550 QTOF instrument (Agilent) in negative mode 4 GHz, high resolution in a m/z range of 50-1000 [32]. A 60:40 mixture of isopropanol:water supplemented with NH_4_F at pH 9.0, as well as 10 nM hexakis(1H, 1H, 3H-tetrafluoropropoxy)phosphazine and 80 nM taurocholic acid for online mass calibration. Ions were annotated to metabolites based on exact mass considering [M-H+] and [M + F-] and 0.001 Da mass accuracy using the HMDB v3.0 database [33]. To account for mass differences of the skin samples, metabolite intensities were normalized using quantile normalization [34]. All data analysis was done using Matlab 2014b (The Mathworks).

For correlation analysis of metabolite levels with age, we calculated the Pearson’s correlation between ion intensities and donor’s age [87]. The p-values were corrected for false discovery rate using Storey’s method (q-values) [88]. Ions with |rho| > 0.25 and q < 0.01 were considered significant.

Significantly changing ions between the old and young conditions were identified with a univariate analysis using a two-sample t-test. Multiple testing correction was performed by correcting p-values for false discovery rate as described before (q-values) [88]. Ions with |log_2_(fold-change)| > 0.1 and q < 0.05 were considered significant. To identify significantly changing metabolic pathways we performed an enrichment analysis on the univariate analysis results using HMDB metabolic pathway definitions [33]. Significantly changing ions (|log_2_(FC)| > 0.1, q < 0.05) were sorted lowest to highest q-values. The p-values for the enrichment were calculated using a hypergeometric test defined as

$$ {p}_{Ptw}\left( Pt{w}_{Hits}\Big| Tota{l}_{AllDetected}, Pt{w}_{AllDetected}, Tota{l}_{Hits}\right) = \frac{\left(\begin{array}{c}\hfill Pt{w}_{AllDetected}\hfill \\ {}\hfill Pt{w}_{Hits}\hfill \end{array}\right)\left(\begin{array}{c}\hfill Tota{l}_{AllDetected}- Pt{w}_{AllDetected}\hfill \\ {}\hfill Tota{l}_{Hits}- Pt{w}_{Hits}\hfill \end{array}\right)}{\left(\begin{array}{c}\hfill Tota{l}_{AllDetected}\hfill \\ {}\hfill Tota{l}_{Hits}\hfill \end{array}\right)} $$

where *Total*_*Hits*_ is the total number of ions in the hit subset, *Total*_*AllDetected*_ is the total number of detected ions (background), *Ptw*_*Hits*_ is the intersect of all ions in the hit subset and ions involved in a given pathway and *Ptw*_*AllDetected*_ is the intersect of all ions and ions involved in a given pathway. The hit subset for each pathway and each comparison between two conditions was defined recursively by first considering only the most significant ion, and then increasing the hit subset with the next best significant ion at each iteration until all significant ions were in the hit subset. Enrichment analysis was performed on each of those hit subsets and the best p-value was used as p-value for the enrichment of a given comparison and a given pathway. We corrected the p-values as described before for multiple testing by Storey's method [88].

### Transcriptomic analysis

Microarray analysis and data extraction was performed using Agilent Whole Human Genome Oligo Microarrays 8x60K V2 and Agilent Feature Extraction Software (Agilent Technologies, Waldbronn, Germany) by Genomics Services from Miltenyi Biotec (Bergisch Gladbach, Germany). Raw data was preprocessed and analyzed using the limma package from bioconductor for R [89]. We removed features that had in at least 50% of the cases either a saturated signal or a signal not distinguishable of the background noise. To account for illumination differences of the different microarrays, the feature intensities of each microarray were normalized using quantile normalization [34]. Differential gene expression was determined using linear models with young and old groups as variables [89]. The p-values were corrected for false-discovery rate using the Benjamini-Hochberg approach [90]. Transcripts with Benjamini-Hochberg adjusted *p* < 0.01 and |log_2_(FC)| > 0.25 were considered significant.

To identify metabolic pathways with significantly overrepresented transcript changes we performed an enrichment analysis on the differentially expressed genes using KEGG metabolic pathway definitions specific for homo sapiens (hsa) [49]. Significantly changing transcripts (Benjamini-Hochberg adjusted *p* < 0.01, |log_2_(FC)| > 0.25) were sorted lowest to highest p-values. The p-values for the enrichment were calculated using a hypergeometric test defined as

$$ {p}_{Ptw}\left( Pt{w}_{Hits}\Big| Tota{l}_{AllDetected}, Pt{w}_{AllDetected}, Tota{l}_{Hits}\right) = \frac{\left(\begin{array}{c}\hfill Pt{w}_{AllDetected}\hfill \\ {}\hfill Pt{w}_{Hits}\hfill \end{array}\right)\left(\begin{array}{c}\hfill Tota{l}_{AllDetected}- Pt{w}_{AllDetected}\hfill \\ {}\hfill Tota{l}_{Hits}- Pt{w}_{Hits}\hfill \end{array}\right)}{\left(\begin{array}{c}\hfill Tota{l}_{AllDetected}\hfill \\ {}\hfill Tota{l}_{Hits}\hfill \end{array}\right)} $$

where *Total*_*Hits*_ is the total number of genes in the hit subset, *Total*_*AllDetected*_ is the total number of detected genes (background), *Ptw*_*Hits*_ is the intersect of all genes in the hit subset and genes involved in a given pathway and *Ptw*_*AllDetected*_ is the intersect of all genes and genes involved in a given pathway. The hit subset for each pathway and each comparison between two conditions was defined recursively by first considering only the most significant genes, and then increasing the hit subset with the next best significant gene at each iteration until all significant genes were in the hit subset. Enrichment analysis was performed on each of those hit subsets and the best p-value was used as p-value for the enrichment of a given comparison and a given pathway. We corrected the p-values as described before for multiple testing by Storey's method (q-values) [88]. Pathways with q-values < 0.01 were considered significantly enriched.

### Integration of transcriptome and metabolome data using locality analysis

The integration of transcriptomics and metabolomics data we performed using the previously described locality analysis on matched transcriptome and metabolome data of 23 young and 23 old human donors [60]. The algorithm scores enzymes according to the weighted sum of the spearman correlation of their gene expression with the levels of surrounding metabolites. Thereby the correlations are weighted according to the distance of the metabolite to the enzyme within the metabolic network. We used the KEGG main reaction pair network specific for *homo sapiens* generated with a modified version of the MetaboNetworks toolbox [91] as metabolic model for the algorithm. Moreover, we reannotated the ions from the metabolomics dataset to metabolites defined in the KEGG hsa database to fit the metabolic model [49]. The locality scores *S*(*t*_*i*_) for a given metabolic enzyme coding transcript *t*_*i*_ are calculated with

$$ S\left({t}_i\right)=\frac{{\displaystyle {\sum}_{m=1}^M}{D}_{i, m}^{-2}\cdot \left(1-{p}_{C_{i, m}}\right)\cdot \left|{C}_{i, m}\right|}{{\displaystyle {\sum}_{m=1}^M}{D}_{i, m}^{-2}\cdot \left(1-{p}_{C_{i, m}}\right)}, $$

where i is the index for the transcripts t, m is the index for metabolites, *C*_*i*,*m*_ is the spearman correlation between the transcripts and the metabolite levels, *D*_*i*,*m*_ is the network distance between metabolite m and transcript *t*_*i*_ and ${p}_{C_{i, m}}$ is the p-value for the spearman correlation between the transcript and metabolite levels. The significance of the final score was determined by comparing the real score to the score of 10000 random locality scores *S*_*rand*_^*k*^(*t*_*i*_) calculated using randomly permuted versions of the distance matrix *D* (*D*_*rand*_). In detail the randomly permuted scores are calculated with

$$ {S}_{ran d}^k\left({t}_i\right) = \frac{{\displaystyle {\sum}_{m=1}^M}{\left({D}_{ran{ d}_{i, m}}\right)}^{-2}\cdot \left(1-{p}_{C_{i, m}}\right)\cdot \left|{C}_{i, m}\right|}{{\displaystyle {\sum}_{m=1}^M}{\left({D}_{ran{ d}_{i, m}}\right)}^{-2}\cdot \left(1-{p}_{C_{i, m}}\right)}, $$

where k is the index of the permutation. The p-value *p*(*S*(*t*_*i*_)) for the locality score of a given transcript *S*(*t*_*i*_) gets calculated with

$$ p\left( S\left({t}_i\right)\right)=\frac{{\displaystyle {\sum}_k}\left({S}_{rand}^k\left({t}_i\right)\ge S\left({t}_i\right)\right)}{K}, $$

where K is the total number of permutations (10000 in this study). Locality scores with *p* < 0.05 were considered significant. Genes with significant locality scores were reduced to filtering them for transcripts with age-dependent changes (*p* < 0.1 of a Spearman’s correlation comparing gene expression and donor age or Benjamini-Hochberg corrected *p* < 0.1 of a univariate analysis comparing transcripts from young and old donors).

## Additional files

Additional file 1: Details on epidermal tissue donors. x indicates that sample were included in metabolomics, transcriptomic or the integrated analysis, respectively. (XLSX 15 kb)
Additional file 2: Quantile normalized non-targeted metabolomics data of epidermal tissue samples of young and old donors. (XLS 1394 kb)
Additional file 3: A) Differential analysis and B) metabolic pathway enrichment results comparing metabolite data from old and young donors. Metabolic changes with log_2_(FC) > 0.1 and q-values < 0.01 were considered significant. Q-values are false-discovery corrected p-values of a t-test. (PDF 617 kb)
Additional file 4: Comparison of predicted and reported significant changing genes during aging. None of the overlaps is significant (hypergeometric test). Data sources: HAGR: Human Aging Genomic Resources [46], Glass et al [47], Makrantonaki et al [19]. (PDF 405 kb)
Additional file 5: Adaptations in gene expression comparing young and old skin. A) Amino- and nucleotidesugar metabolism, B) inositol phosphate metabolism, C) aminoacyl t-RNA biosynthesis, D) arginine and proline metabolism and E) retinol metabolism. Significant transcript changes (adj.*p* < 0.01 and |log2(Old/Young)| > 0.25) are marked with a star *. (PDF 526 kb)
Additional file 6: Results of the locality analysis. The results for all enzymes with p-value < 0.05 in the locality analysis are shown. (PDF 492 kb)
Additional file 7: Locality analysis results of the Aldehyde Dehydrogenase 4 Family, Member A1 (ALDH4A1). Upper panel: Locality scores for and metabolite-gene correlation. Lower panel: Metabolite-age and gene-age correlation. Node size indicates significance. (PDF 419 kb)
Additional file 8: Locality analysis results of Branched Chain Keto Acid Dehydrogenase E1 (BCKDHA) in valine, leucine and isoleucine metabolism. Upper panel: Locality scores for and metabolite-gene correlation. Lower panel: Metabolite-age and gene-age correlation. Node size indicates significance. (PDF 435 kb)
Additional file 9: Locality analysis results of the Interleukin 4 Induced (IL4I1), a lysosomal L-amino-acid oxidase. Upper panel: Locality scores for and metabolite-gene correlation. Lower panel: Metabolite-age and gene-age correlation. Node size indicates significance. (PDF 442 kb)
Additional file 10: Code for data analysis Matlab and R code to perform the gene expression and locality analysis performed in this manuscript. (ZIP 36968 kb)

## Abbreviations

AGPAT9: Glycerol-3-phosphate acyltransferase 3
ALDH4A1: Aldehyde dehydrogenase 4 family, member A1
ALDOA: Aldolase A
BCKDHA: Branched chain keto acid dehydrogenase
CoA: Coenzyme A
DHEA: Dehydroepiandrosterone
FBP1: Bisphosphatase 1
FDR: False discovery rate
GAPDH: Glyceraldehyde 3-phosphate dehydrogenase
GK: Glycerol kinase
GLS: Glutaminase
HK2: Hexokinase 2
HMDB: Human Metabolome Database
hsa: Homo sapiens
HSD11B2: Hydroxysteroid (11-beta) dehydrogenase 2
IL4I1: Interleukin 4 induced 1
IMPA2: Inositol-1(or 4)-monophosphatase 2
KEGG: Kyoto Encyclopedia of Genes and Genomes
mTOR: Mammalian target of rapamycin
ODC1: Ornithine decarboxylase 1
PFKP: Phosphofructokinase
UV: Ultraviolet
