# Supplementary figures and images for: An integrative metabolomics and transcriptomics study to identify metabolic alterations in aged skin of humans in vivo
Source: BMC Genomics. 2017 Feb 15;18:169. doi: 10.1186/s12864-017-3547-3 (PMC5312537; doi:10.1186/s12864-017-3547-3)

A

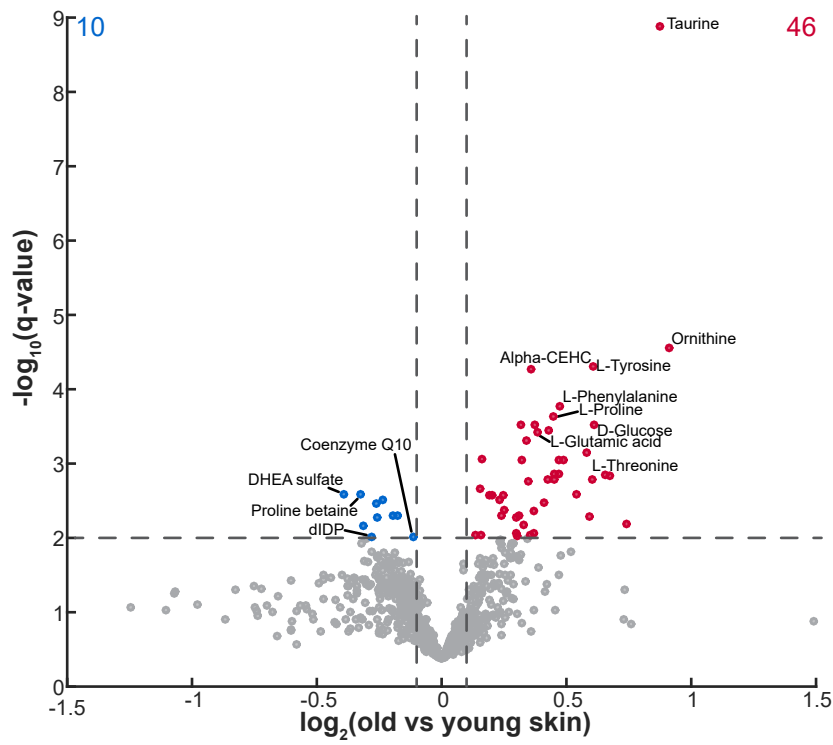

B

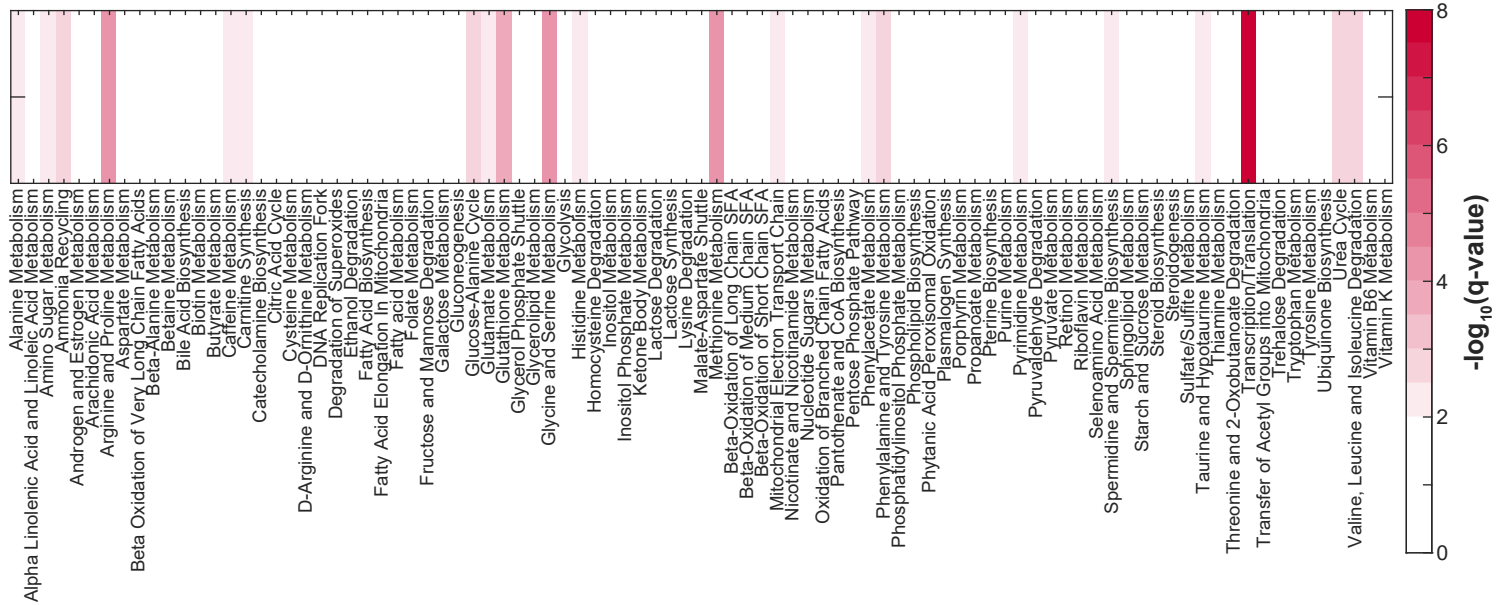

Supplement: Additional file 3: — A) Differential analysis and B) metabolic pathway enrichment results comparing metabolite data from old and young donors. Metabolic changes with log2(FC) > 0.1 and q-values < 0.01 were considered significant. Q-values are false-discovery corrected p-values of a t-test. (PDF 617 kb) [file 12864_2017_3547_MOESM3_ESM.pdf]

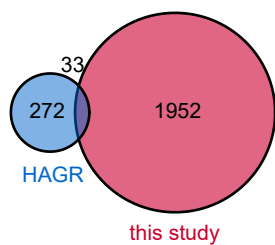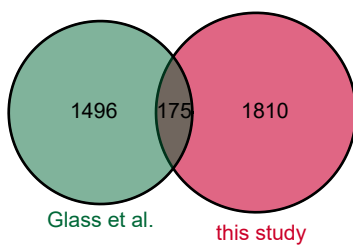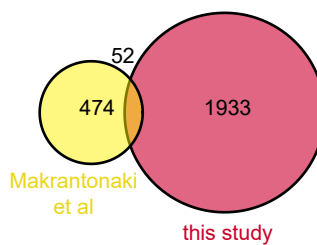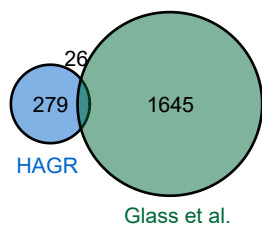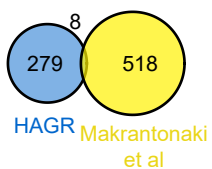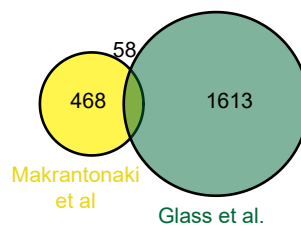

Supplement: Additional file 4: — Comparison of predicted and reported significant changing genes during aging. None of the overlaps is significant (hypergeometric test). Data sources: HAGR: Human Aging Genomic Resources [46], Glass et al [47], Makrantonaki et al [19]. (PDF 405 kb) [file 12864_2017_3547_MOESM4_ESM.pdf]

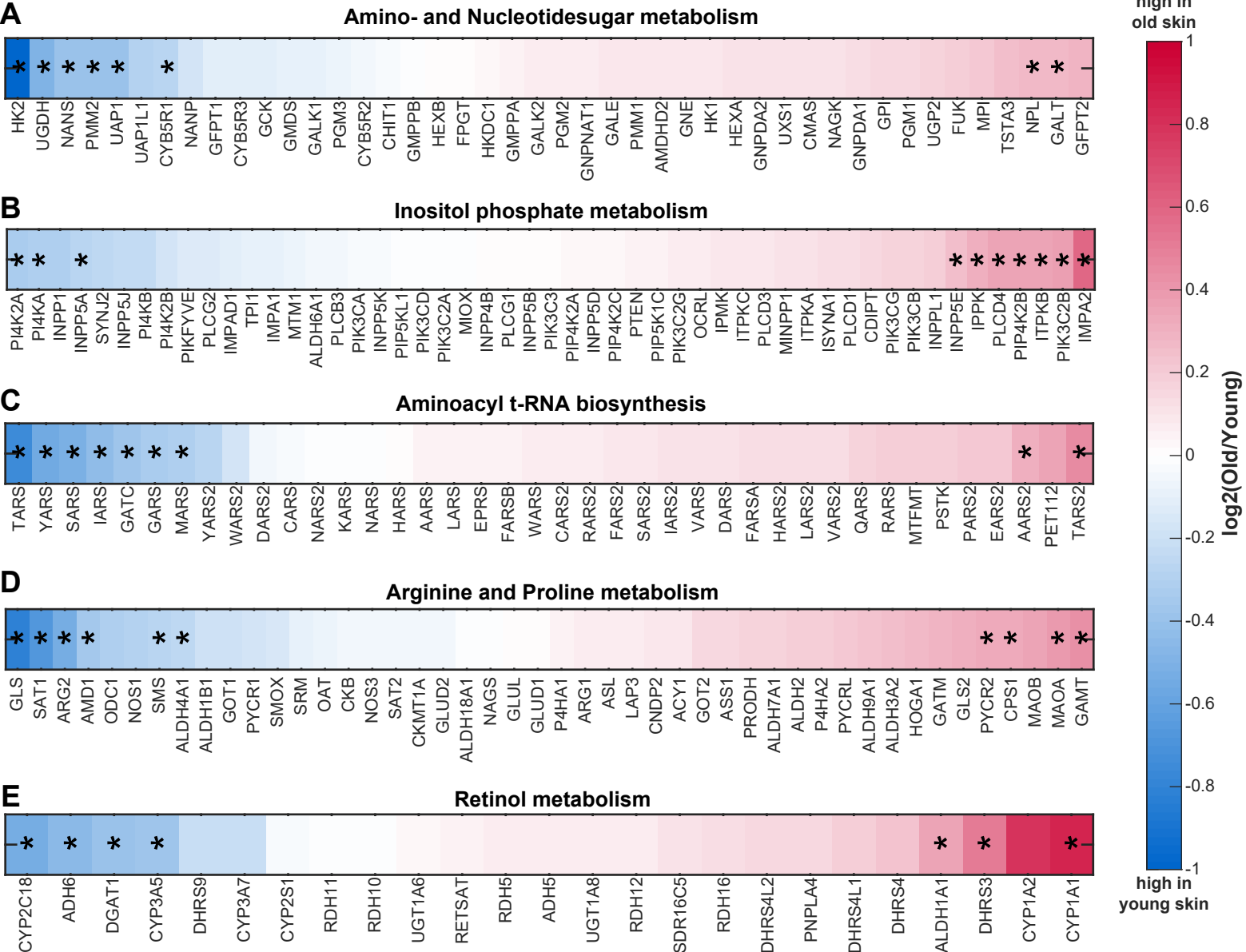

Supplement: Additional file 5: — Adaptations in gene expression comparing young and old skin. A) Amino- and nucleotidesugar metabolism, B) inositol phosphate metabolism, C) aminoacyl t-RNA biosynthesis, D) arginine and proline metabolism and E) retinol metabolism. Significant transcript changes (adj.p < 0.01 and |log2(Old/Young)| > 0.25) are marked with a star *. (PDF 526 kb) [file 12864_2017_3547_MOESM5_ESM.pdf]

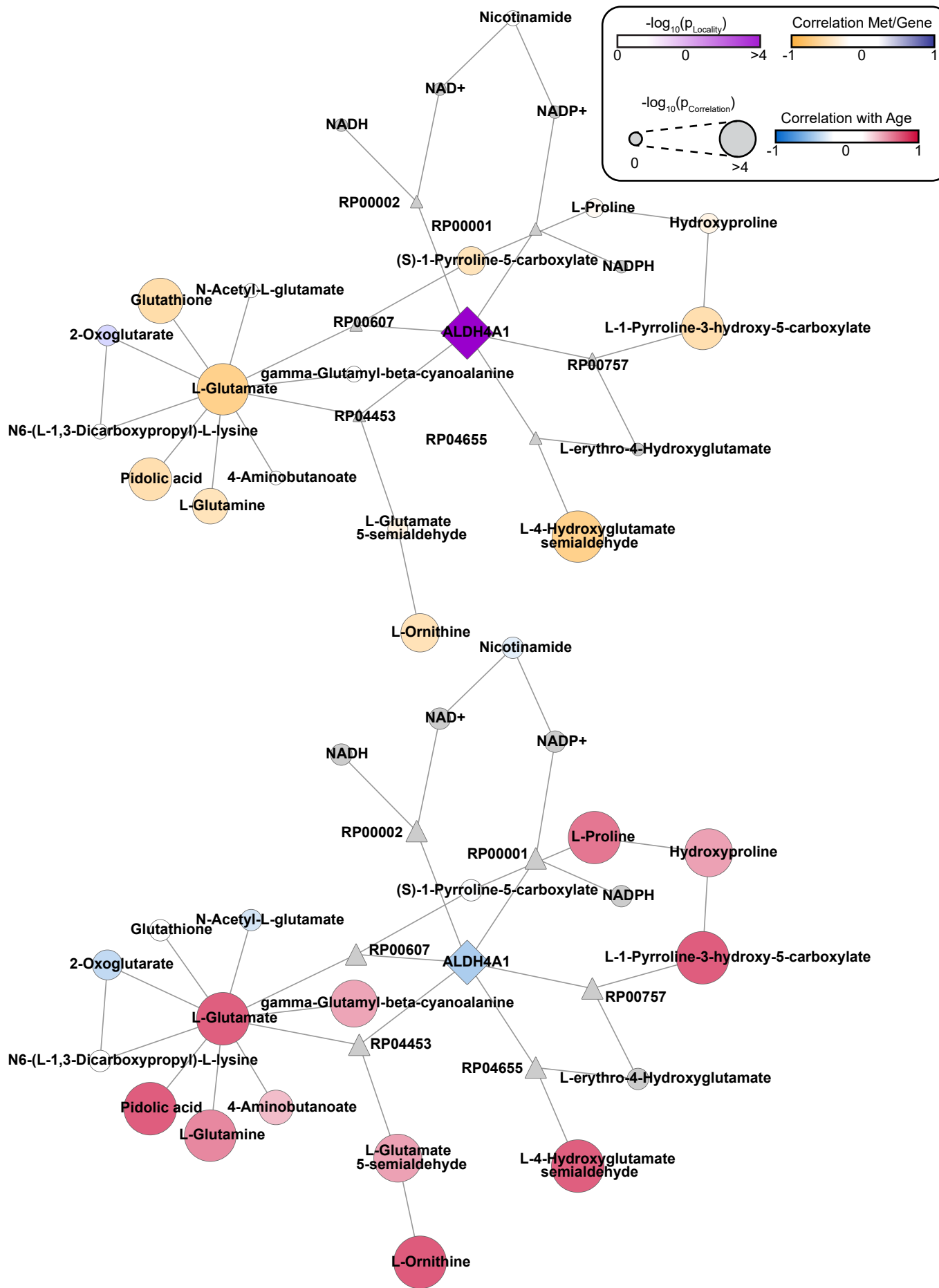

Supplement: Additional file 7: — Locality analysis results of the Aldehyde Dehydrogenase 4 Family, Member A1 (ALDH4A1). Upper panel: Locality scores for and metabolite-gene correlation. Lower panel: Metabolite-age and gene-age correlation. Node size indicates significance. (PDF 419 kb) [file 12864_2017_3547_MOESM7_ESM.pdf]

# Locality analysis

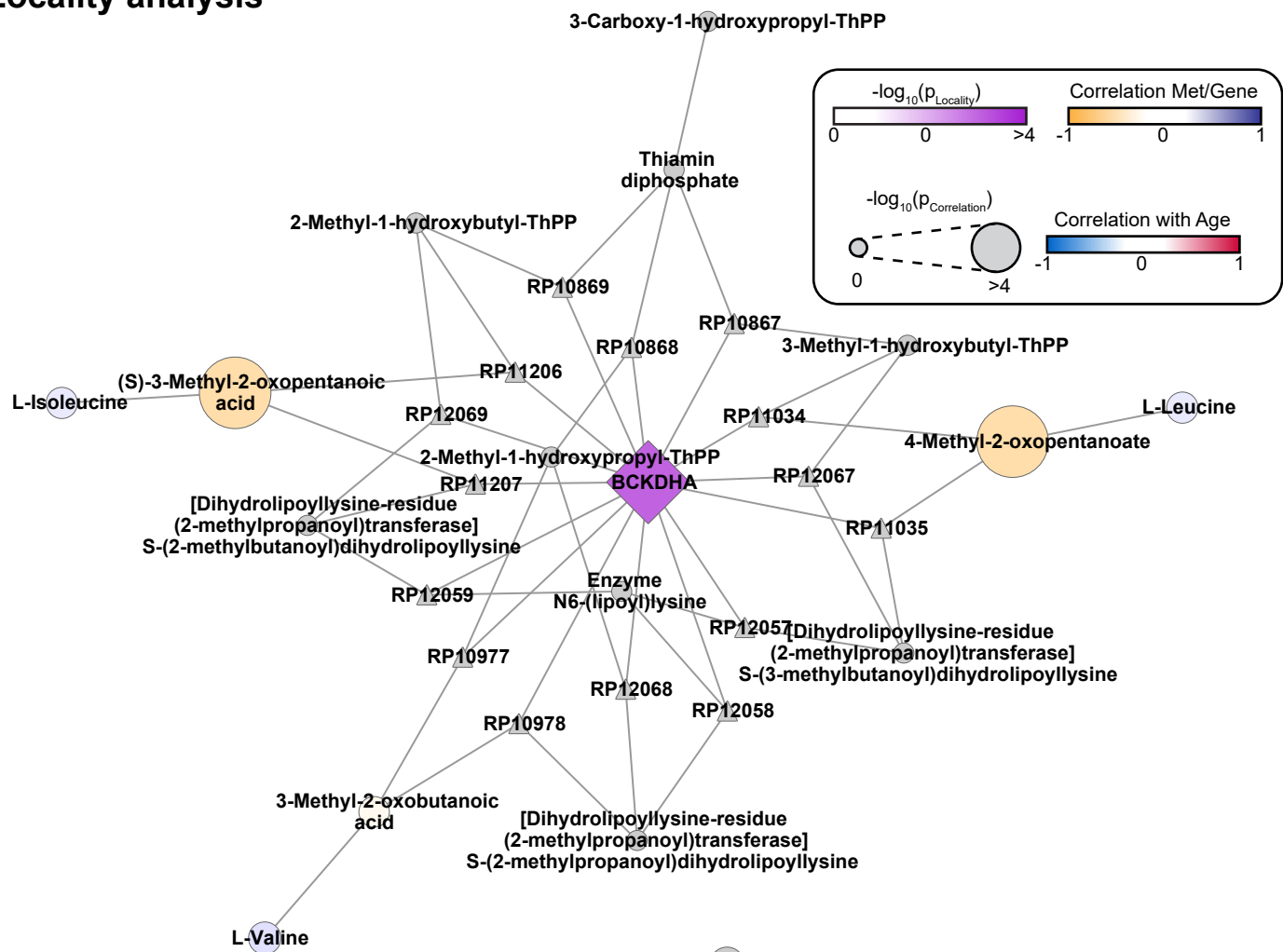

# Age correlation

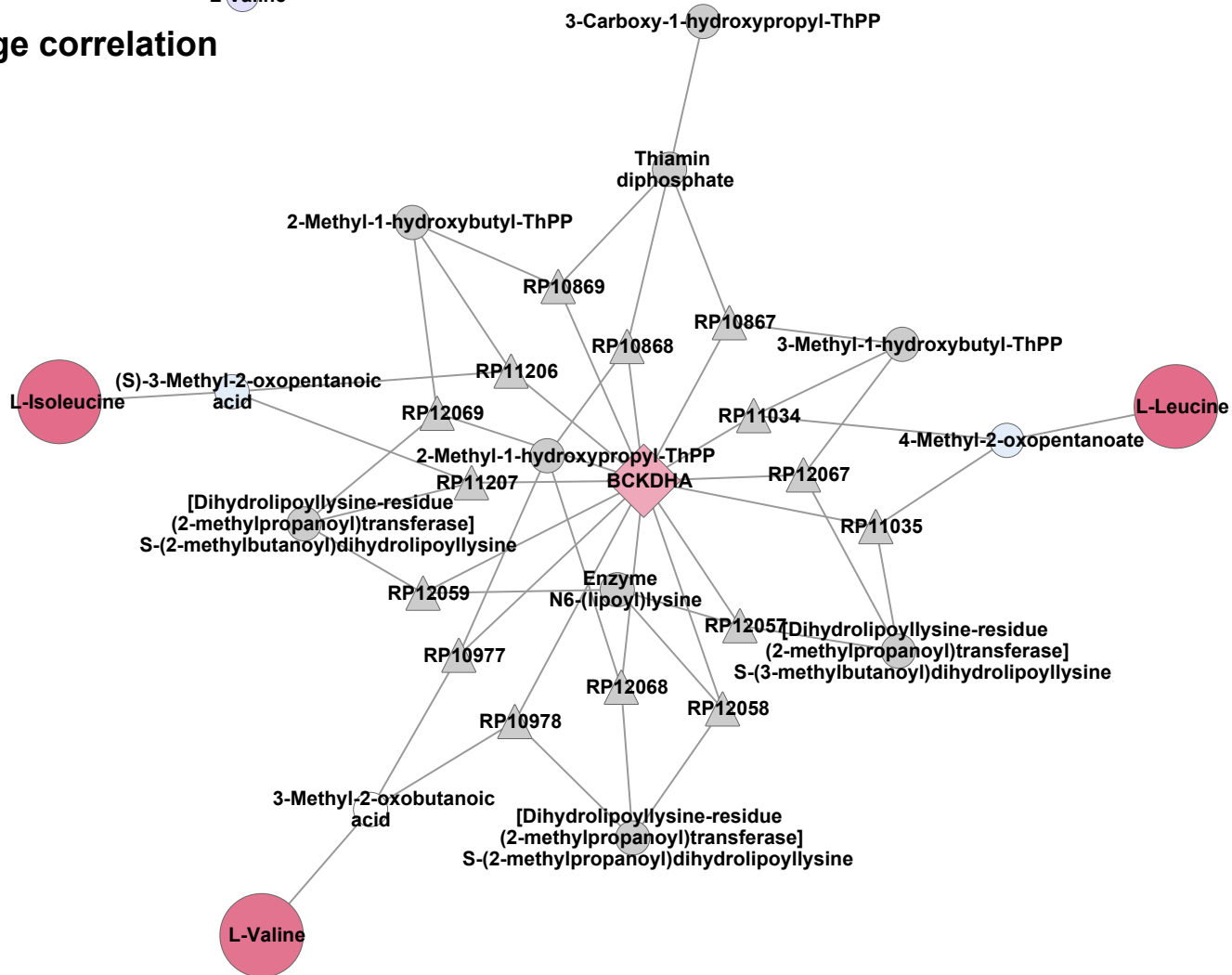

Supplement: Additional file 8: — Locality analysis results of Branched Chain Keto Acid Dehydrogenase E1 (BCKDHA) in valine, leucine and isoleucine metabolism. Upper panel: Locality scores for and metabolite-gene correlation. Lower panel: Metabolite-age and gene-age correlation. Node size indicates significance. (PDF 435 kb) [file 12864_2017_3547_MOESM8_ESM.pdf]

## Locality analysis

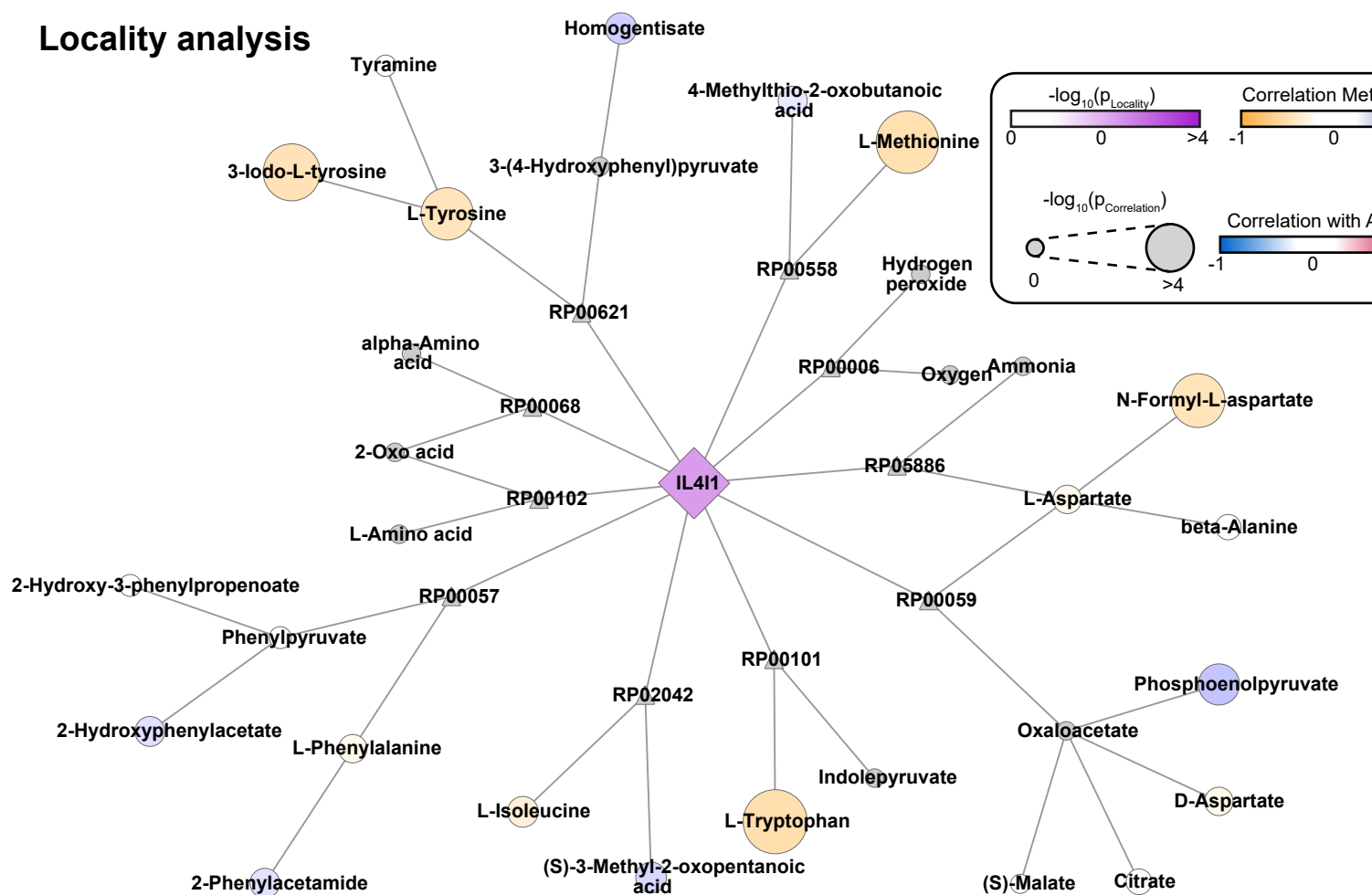

## Age correlation

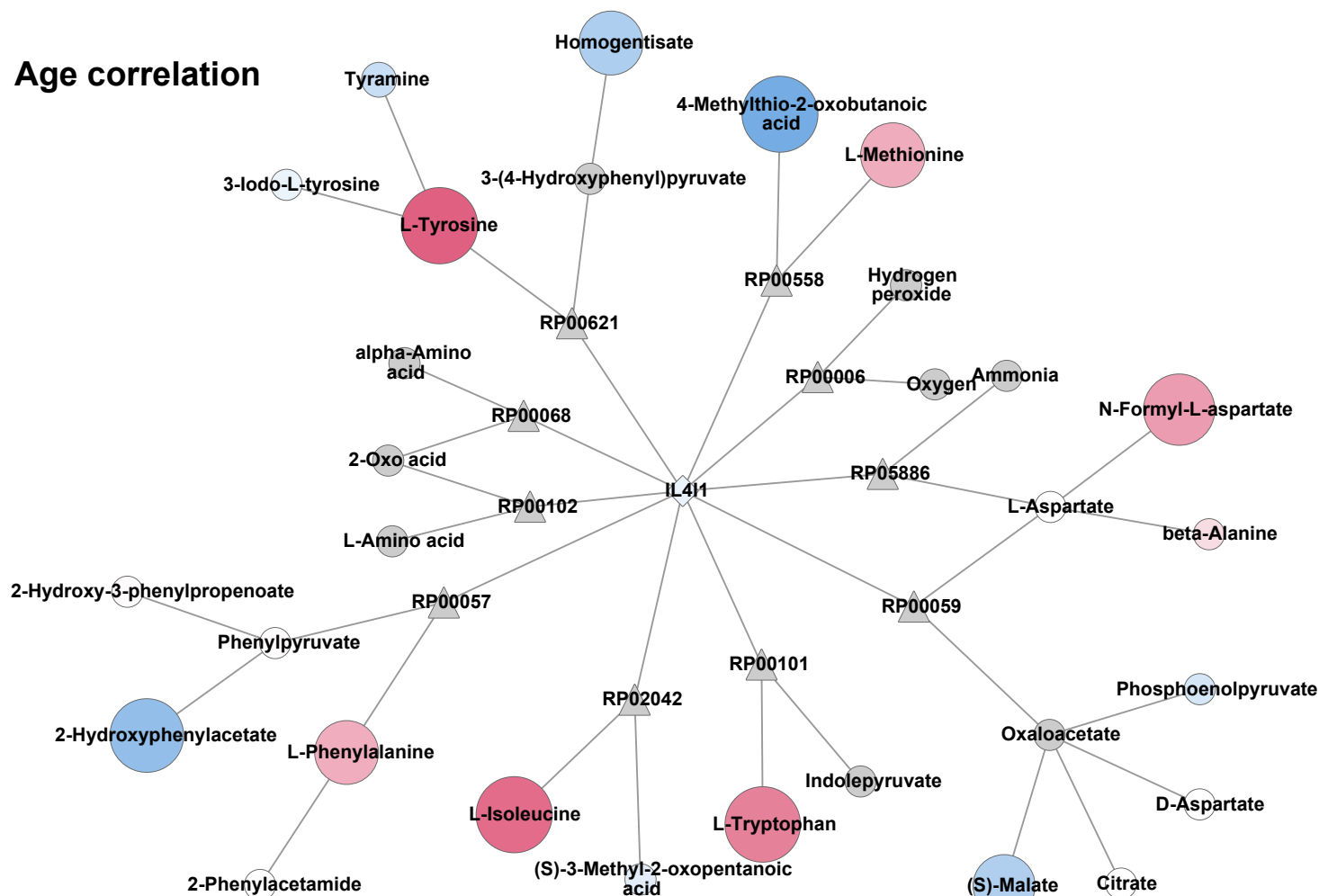

Supplement: Additional file 9: — Locality analysis results of the Interleukin 4 Induced (IL4I1), a lysosomal L-amino-acid oxidase. Upper panel: Locality scores for and metabolite-gene correlation. Lower panel: Metabolite-age and gene-age correlation. Node size indicates significance. (PDF 442 kb) [file 12864_2017_3547_MOESM9_ESM.pdf]
