# Supplementary material for: An integrative metabolomics and transcriptomics study to identify metabolic alterations in aged skin of humans in vivo
Source: BMC Genomics. 2017 Feb 15;18:169. doi: 10.1186/s12864-017-3547-3 (PMC5312537; doi:10.1186/s12864-017-3547-3)
Supplement: Additional file 6: — Results of the locality analysis. The results for all enzymes with p-value < 0.05 in the locality analysis are shown. (PDF 492 kb) [file 12864_2017_3547_MOESM6_ESM.pdf]

Rank	Gene Symbol	Gene Name	Locality		Correlation Gene/Age		Diff. analysis Old/young	
			Score	p	r	p	log_2 (FC)	adj.p
1	VKORC1	vitamin K epoxide reductase complex, subunit 1	0.24	0	0.04	0.79341	0.13	0.48027
2	ALDH4A1	aldehyde dehydrogenase 4 family, member A1	0.25	0.0001	-0.33	0.02619	-0.26	0.00638
3	ODC1	ornithine decarboxylase 1	0.26	0.0002	-0.26	0.07619	-0.30	0.08512
4	PARP1	poly (ADP-ribose) polymerase 1	0.30	0.0004	0.12	0.42495	0.06	0.54265
5	GALNT6	polypeptide N-acetylgalactosaminyltransferase 6	0.22	0.0004	0.38	0.00963	0.30	0.11179
6	CHPF2	chondroitin polymerizing factor 2	0.20	0.0005	0.08	0.59005	0.15	0.1742
7	CYP4F3	cytochrome P450, family 4, subfamily F, polypeptide 3	0.20	0.0006	0.08	0.59403	0.02	0.91227
8	TARS2	threonyl-tRNA synthetase 2, mitochondrial (putative)	0.31	0.0007	0.55	6.6E-05	0.45	7.1E-05
9	BCKDHA	branched chain keto acid dehydrogenase E1, alpha polypeptide	0.21	0.0023	0.34	0.0216	0.18	0.02631
10	ALDOA	aldolase A, fructose-bisphosphate	0.24	0.0029	0.57	3.3E-05	0.24	0.00189
11	TARS	threonyl-tRNA synthetase	0.29	0.0029	-0.54	0.00011	-0.75	6.3E-06
12	SRD5A3	steroid 5 alpha-reductase 3	0.20	0.0035	0.12	0.42737	0.10	0.3851
13	PANK4	pantothenate kinase 4	0.21	0.0048	-0.22	0.14847	-0.14	0.14408
14	YARS2	tyrosyl-tRNA synthetase 2, mitochondrial	0.28	0.006	-0.39	0.00726	-0.27	0.01066
15	UPRT	uracil phosphoribosyltransferase (FUR1) homolog (S. cerevisiae)	0.21	0.0075	-0.20	0.17793	-0.09	0.14608
16	CYP51A1	cytochrome P450, family 51, subfamily A, polypeptide 1	0.19	0.0079	-0.40	0.00549	-0.20	0.01213
17	ALAD	aminolevulinate dehydratase	0.24	0.0081	0.19	0.20379	0.22	0.06121
18	PTGDS	prostaglandin D2 synthase 21kDa (brain)	0.23	0.0094	0.22	0.14587	0.11	0.83769
19	LIAS	lipoic acid synthetase	0.23	0.0099	0.23	0.12402	0.13	0.2205
20	G6PD	glucose-6-phosphate dehydrogenase	0.20	0.0109	-0.13	0.4011	0.00	0.97172
21	AKR1A1	aldo-keto reductase family 1, member A1 (aldehyde reductase)	0.18	0.0129	0.17	0.26891	0.07	0.56564
22	SMOX	spermine oxidase	0.21	0.0129	-0.02	0.8961	-0.16	0.60183
23	ALDOC	aldolase C, fructose-bisphosphate	0.18	0.0132	-0.01	0.92206	0.17	0.36329
24	ACOT7	acyl-CoA thioesterase 7	0.20	0.0134	-0.15	0.33452	-0.05	0.70997
25	IL4I1	interleukin 4 induced 1	0.21	0.014	-0.16	0.29659	-0.58	0.05193
26	POLE2	polymerase (DNA directed), epsilon 2, accessory subunit	0.30	0.0154	0.19	0.19815	0.13	0.16433
27	FBP1	fructose-1,6-bisphosphatase 1	0.27	0.0164	0.34	0.02183	0.22	0.09394
28	GATM	glycine amidinotransferase (L-arginine:glycine amidinotransferase)	0.26	0.0172	0.37	0.01177	0.29	0.01911
29	HAL	histidine ammonia-lyase	0.22	0.0174	-0.07	0.63096	0.08	0.82855
30	P4HA1	prolyl 4-hydroxylase, alpha polypeptide I	0.19	0.0193	0.12	0.43976	0.06	0.67736
31	YARS	tyrosyl-tRNA synthetase	0.27	0.0198	-0.48	0.00081	-0.54	0.00065
32	GART	phosphoribosylglycinamide formyltransferase, phosphoribosylglycinamide synthetase,	0.28	0.0205	-0.35	0.01882	-0.39	0.00604
33	PRDX6	peroxiredoxin 6	0.24	0.0205	0.36	0.01457	0.14	0.0556
34	ITPKC	inositol-trisphosphate 3-kinase C	0.17	0.0216	0.12	0.42492	0.09	0.6661
35	IRAK2	interleukin-1 receptor-associated kinase 2	0.17	0.0217	0.04	0.78348	0.02	0.92987
36	GGT6	gamma-glutamyltransferase 6	0.19	0.0242	0.12	0.41684	0.21	0.1193
37	LSS	lanosterol synthase (2,3-oxidosqualene-lanosterol cyclase)	0.20	0.0269	-0.20	0.17402	-0.05	0.85824
38	DCT	dopachrome tautomerase	0.22	0.0272	0.21	0.16638	0.33	0.25858
39	AHCYL1	adenosylhomocysteinase-like 1	0.19	0.0278	-0.02	0.87217	0.05	0.55199
40	GPT	glutamic-pyruvate transaminase (alanine aminotransferase)	0.22	0.028	-0.04	0.80497	0.03	0.82611
41	GGCT	gamma-glutamylcyclotransferase	0.22	0.0293	-0.08	0.59261	0.03	0.907
42	AGA	aspartylglucosaminidase	0.18	0.0319	0.25	0.10067	0.15	0.07723
43	TST	thiosulfate sulfurtransferase (rhodanese)	0.24	0.0339	0.31	0.03907	0.31	0.04075
44	DCXR	dicarbonyl/L-xylulose reductase	0.16	0.0351	-0.22	0.14328	-0.07	0.43955
45	PGM2	phosphoglucomutase 2	0.21	0.0375	0.10	0.50065	0.07	0.53554
46	ACP5	acid phosphatase 5, tartrate resistant	0.26	0.0377	0.31	0.0356	0.36	0.00665
47	CYP39A1	cytochrome P450, family 39, subfamily A, polypeptide 1	0.22	0.0377	0.00	0.98481	0.03	0.86186
48	ASAH2	N-acylsphingosine amidohydrolase (non-lysosomal ceramidase) 2	0.16	0.0379	0.22	0.15051	0.05	0.53135
49	IARS	isoleucyl-tRNA synthetase	0.28	0.0386	-0.40	0.00582	-0.44	0.00156
50	FUT3	fucosyltransferase 3 (galactoside 3(4)-L-fucosyltransferase, Lewis blood group)	0.19	0.0408	0.08	0.58863	0.14	0.3985
51	NME4	NME/NM23 nucleoside diphosphate kinase 4	0.18	0.0409	-0.11	0.46471	-0.03	0.73687

Rank	Gene Symbol	Gene Name	Locality		Correlation Gene/Age		Diff. analysis Old/young	
			Score	p	r	p	log_2 (FC)	adj.p
52	NDUFV2	NADH dehydrogenase (ubiquinone) flavoprotein 2, 24kDa	0.22	0.0412	0.26	0.07692	0.13	0.06231
53	SGPL1	sphingosine-1-phosphate lyase 1	0.17	0.0413	0.05	0.75373	0.16	0.11062
54	MPST	mercaptopyruvate sulfurtransferase	0.20	0.0429	0.18	0.23438	0.11	0.32854
55	ND3	NADH dehydrogenase, subunit 3 (complex I)	0.17	0.0451	-0.17	0.25676	-0.12	0.39405
56	ALDH1B1	aldehyde dehydrogenase 1 family, member B1	0.27	0.0453	-0.14	0.34069	-0.19	0.18115
57	ALPPL2	alkaline phosphatase, placental-like 2	0.20	0.0461	-0.10	0.50934	-0.05	0.672
58	AASS	aminoadipate-semialdehyde synthase	0.17	0.0463	-0.19	0.2119	-0.15	0.19581
59	IDH3B	isocitrate dehydrogenase 3 (NAD+) beta	0.19	0.0478	-0.09	0.5329	0.00	0.9736
60	FASN	fatty acid synthase	0.18	0.0479	0.06	0.70595	0.04	0.67724
61	PDHA1	pyruvate dehydrogenase (lipoamide) alpha 1	0.20	0.0496	-0.17	0.27044	-0.07	0.28241
