# Supplementary material for: An integrative metabolomics and transcriptomics study to identify metabolic alterations in aged skin of humans in vivo
Source: BMC Genomics. 2017 Feb 15;18:169. doi: 10.1186/s12864-017-3547-3 (PMC5312537; doi:10.1186/s12864-017-3547-3)
Supplement: Additional file 10: — Code for data analysis Matlab and R code to perform the gene expression and locality analysis performed in this manuscript. (ZIP 36968 kb) [file 12864_2017_3547_MOESM10_ESM.zip › Data analysis - Read me.pdf]

Data analysis – Read me

This folder contains computational scripts for data analysis performed in the manuscript "Metabolic alterations in aged human skin *in vivo*" by Kuehne and Hildebrand *et al*.

Analysis of gene expression data

For the analysis of the gene expression comparing epidermal samples of old and young epidermal donors, download the arrays from gene expression omnibus. The analysis is performed in R and requires the "limma" (https://bioconductor.org/packages/release/bioc/html/limma.html) and "xlsx" (https://cran.r-project.org/web/packages/xlsx/index.html) libraries. Open the "GeneExpression_OldVsYoung_limma_analysis_script.R" script and modify the workingDirectory variable to the path in which you saved the raw data files. Copy the "targets_Array_Age_2.txt" into the raw data folder. Run the script to perform the differential analysis using the limma package.

Integration of metabolomics and transcriptomics data using locality analysis

To identify age-dependent metabolic adaptations that are transcriptionally regulated we applied the locality analysis approach [1] on the integrated metabolomics and transcriptomics data sets, which we performed in Matlab. To run the analysis extract the "Locality analysis" folder and navigate to it in Matlab. Open and run the "metabolic_alterations_in_aged_skin_analysis_script.m".

References

1. Schulz, J.C., et al., *Large-scale functional analysis of the roles of phosphorylation in yeast metabolic pathways.* Sci Signal, 2014. **7**(353): p. rs6.
